# Elastosonography in the Differential Diagnosis of Musculoskeletal Soft Tissue Tumors: A Systematic Review

**DOI:** 10.3390/jcm15020498

**Published:** 2026-01-08

**Authors:** Federica Messina, Antonio Ziranu, Donato Coppola, Mario Di Diego, Giacomo Capece, Consolato Gulli, Fabrizio Termite, Linda Galasso, Maria Assunta Zocco, Giulio Maccauro, Raffaele Vitiello

**Affiliations:** 1 Orthopaedics and Traumatology Department, Università Cattolica del Sacro Cuore, 00168 Roma, Italy; federicamessina695@gmail.com (F.M.); giacomocapece97@gmail.com (G.C.);; 2 Department of Orthopedics, Ospedale Isola Tiberina-Gemelli Isola, 00186 Rome, Italy; 3 Department of Radiological and Hematological Sciences, Università Cattolica del Sacro Cuore, Largo Agostino Gemelli, 10, 00168 Rome, Italy; 4 CEMAD Digestive Diseases Center, Fondazione Policlinico Universitario “Agostino Gemelli” IRCCS, Università Cattolica del Sacro Cuore, Largo Agostino Gemelli, 8, 00168 Rome, Italymariaassunta.zocco@policlinicogemelli.it (M.A.Z.)

**Keywords:** soft tissue tumor, elastography, musculoskeletal tumor, sarcoma, bone tumor, ultrasound, differential diagnosis

## Abstract

**Background:** Soft tissue tumors (STTs) represent a heterogeneous group of rare lesions that frequently mimic bone sarcomas in both clinical and radiologic appearance. Accurate differentiation between benign and malignant lesions is critical for appropriate treatment planning, yet conventional imaging often remains inconclusive. Ultrasound (US) elastography, a non-invasive method that quantifies tissue stiffness, has recently emerged as a potential adjunct to standard musculoskeletal imaging for improving diagnostic confidence and guiding biopsy. **Methods:** A systematic review was conducted in accordance with PRISMA guidelines. PubMed, Web of Science, and Cochrane Library were searched using the keywords “elastography”, “sonoelastography”, and “soft tissue tumor”. Twelve studies encompassing 1554 patients met the inclusion criteria, assessing the diagnostic accuracy of strain, compression, and shear wave elastography for differentiating benign from malignant STTs. **Results:** Elastography alone demonstrated limited specificity when used as a single diagnostic technique. However, its integration into multiparametric ultrasound approaches—combining grayscale, Doppler, and contrast-enhanced imaging—significantly improved diagnostic performance. Several studies reported sensitivities and specificities exceeding 85% when elastographic parameters were incorporated into composite diagnostic scores. **Conclusions:** Ultrasound elastography shows promise as a quantitative imaging biomarker for the preoperative evaluation of musculoskeletal tumors, particularly in distinguishing soft tissue from bone-related lesions. Although not a substitute for histopathological confirmation, its application within multimodal ultrasound protocols may reduce unnecessary biopsies, enhance diagnostic accuracy, and facilitate tailored management of bone and soft tissue sarcomas.

## 1. Introduction

Soft tissue tumors (STTs) encompass a wide and heterogeneous group of neoplasms originating from mesenchymal tissues, including muscle, fat, and fibrous connective structures. Although most STTs are benign, their malignant counterparts—soft tissue sarcomas—pose a considerable diagnostic challenge due to their rarity, variable biological behavior, and overlapping imaging features with benign lesions and even primary bone sarcomas. Accurate preoperative characterization is therefore essential for determining appropriate management strategies and optimizing patient outcomes [1,2,3].

Conventional imaging modalities, such as radiography, computed tomography (CT), and magnetic resonance imaging (MRI), play a fundamental role in tumor assessment but often lack sufficient specificity to reliably distinguish between benign and malignant soft tissue lesions [4,5]. In many cases, histopathologic confirmation through biopsy remains mandatory. However, biopsies can be invasive, technically demanding, and occasionally yield nondiagnostic samples, particularly in deep-seated or heterogeneous lesions. This diagnostic gap has prompted growing interest in the development of non-invasive imaging biomarkers capable of improving tissue characterization before surgery [4,6,7]. Recent advances in multiparametric ultrasound (mpUS) have further highlighted the complementary role of elastography within a comprehensive imaging workflow for soft tissue tumor assessment [8].

Ultrasound (US) represents a cornerstone of initial soft tissue evaluation due to its real-time imaging capabilities, accessibility, and absence of ionizing radiation. The introduction of US elastography—a technique that quantifies tissue stiffness—has added a novel dimension to musculoskeletal imaging [4,7]. Elastography relies on the principle that malignant tumors generally exhibit greater stiffness than benign lesions, and it allows both qualitative and quantitative assessment through strain, compression, or shear wave modalities. When integrated with grayscale and Doppler ultrasound, elastography may enhance diagnostic confidence and provide valuable information for biopsy targeting and surgical planning [9,10,11]. A detailed description of the elastographic principles and methodological approaches adopted in the analyzed studies is provided in the Methods section.

Recent evidence suggests that elastography could serve as a quantitative imaging biomarker in musculoskeletal oncology, potentially bridging the diagnostic continuum between soft tissue and bone tumors [12,13]. This is particularly relevant given the frequent clinical and imaging overlap between soft tissue sarcomas and bone sarcomas, where early and accurate differentiation directly influences surgical approach, adjuvant treatment, and prognosis [14,15]. Therefore, assessing the diagnostic performance of elastosonographic techniques represents a timely and clinically relevant objective in the current landscape of bone and soft tissue tumor management.

The present systematic review aims to critically evaluate the role of ultrasound elastography in differentiating benign from malignant soft tissue tumors, highlighting its potential contribution to the preoperative assessment of musculoskeletal and bone-adjacent lesions. By summarizing current evidence, we aim to clarify the clinical value of elastography within a multiparametric diagnostic framework and to outline its prospective applications in precision oncologic imaging.

## 2. Methods

This systematic review was conducted in accordance with the Preferred Reporting Items for Systematic Reviews and Meta-Analyses (PRISMA) guidelines [12] (see Table S1 in the Supplementary Materials). A comprehensive literature search was performed using the PubMed, Web of Science, and Cochrane Library databases to identify studies evaluating the role of ultrasound (US) elastography in differentiating benign from malignant soft tissue tumors (STTs). The search strategy included combinations of the following terms: “elastography”, “sonoelastography”, “elastosonography”, and “soft tissue tumor” or “sarcoma”. No restriction was applied regarding publication year, language, or study design to ensure maximal coverage of available evidence. In addition, reference lists of included articles were manually screened to identify additional eligible studies not captured in the initial search.

### 2.1. Study Selection

All retrieved records were screened independently by two reviewers (F.M. and D.C.), who evaluated titles, abstracts, and full texts to determine eligibility. Disagreements were resolved by consensus with a third reviewer (R.V.). Inclusion criteria were as follows: (1) studies written in English; (2) detailed description of the elastography technique employed; (3) inclusion of both benign and malignant STTs in the same study cohort; (4) availability of histopathological confirmation; and (5) sufficient quantitative or qualitative data extractable for analysis. Exclusion criteria included: (1) abstracts, reviews, or case reports; (2) duplicate publications; (3) incomplete or missing data; and (4) studies that did not evaluate the same patient group using both elastographic and conventional ultrasound techniques.

**Figure 1:**
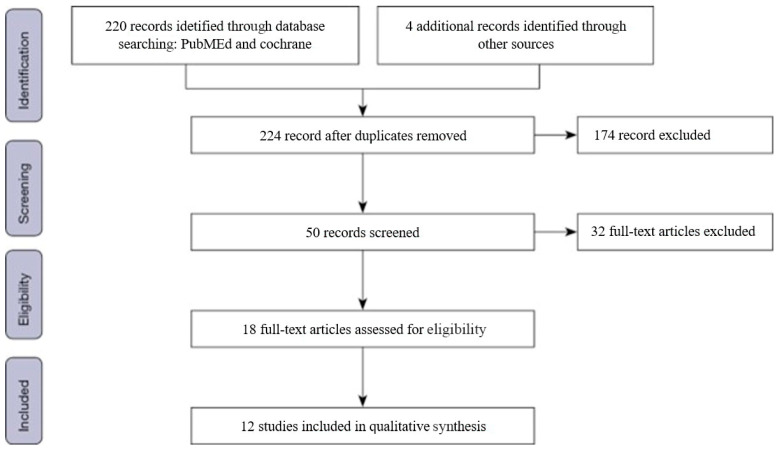
PRISMA 2020 flow diagram illustrating the study selection process for this systematic review. A total of 224 records were identified after duplicate removal. Following title and abstract screening, 50 studies were assessed in full text, and 12 met the predefined inclusion criteria and were included in the qualitative synthesis. The flowchart summarizes the identification, screening, eligibility, and inclusion stages in accordance with PRISMA guidelines [12].

A PRISMA flow diagram illustrating the selection process is presented in Figure 1.

### 2.2. Data Extraction and Analysis

For each included study, the following data were extracted: authors, publication year, study design, number of participants, elastographic modality used (strain, compression, or shear wave elastography), diagnostic parameters evaluated, and main outcomes. Quantitative metrics such as shear wave velocity (SWV) or strain ratio (SR) were recorded whenever available. Qualitative scoring systems—including the Tsukuba Elasticity Score, Itoh score, and Park grading scale—were also noted [13,14,15,16].

Due to the heterogeneity of study designs, populations, and statistical methods, a quantitative meta-analysis was not feasible. Therefore, a narrative synthesis was conducted to summarize trends, highlight methodological differences, and compare diagnostic performances across elastographic modalities.

### 2.3. Technical Principles of Elastography

US elastography is a non-invasive imaging technique that evaluates tissue stiffness based on mechanical response to applied stress. The underlying principle follows Hooke’s law, which relates tissue elasticity (E) to the ratio between applied stress and resulting strain (E = stress/strain) [17].

Compression elastography (CE) and strain elastography (SE) are semi-quantitative, operator-dependent modalities that rely on manual compression. In these methods, tissue stiffness is expressed as a strain ratio between the target lesion and adjacent reference tissue, typically subcutaneous fat [4,7].

Shear wave elastography (SWE), by contrast, is a quantitative and less operator-dependent technique that employs acoustic radiation force to generate shear waves. The propagation velocity of these waves (expressed in meters per second) or the derived shear modulus reflects tissue stiffness and correlates with potential malignancy [9,10,11,18].

Integrating elastography with conventional grayscale and Doppler ultrasound may improve lesion characterization, particularly in complex or equivocal cases, and assist in directing biopsy to the most representative tumor area [19,20].

### 2.4. Quality Assessment

Methodological quality and risk of bias of included studies were assessed according to PRISMA recommendations and previously validated quality appraisal frameworks [12,21]. Any discrepancies in evaluation were resolved by discussion among the authors until consensus was achieved.

#### Use of Generative AI

Generative artificial intelligence tools (ChatGPT, GPT-5, OpenAI, San Francisco, CA, USA) were used to improve the English language and clarity of the text. All content was reviewed and approved by the authors, who take full responsibility for the final manuscript.

## 3. Results

### 3.1. Study Selection Results

From an initial pool of 224 identified studies, a stepwise selection process was conducted in accordance with PRISMA guidelines [12]. After removing duplicates and screening titles and abstracts, 50 full-text articles were evaluated for eligibility. Ultimately, 12 studies met the predefined inclusion criteria and were included in this review (Figure 1).

The analyzed cohort comprised a total of 1554 patients with 1563 histologically confirmed soft tissue tumors. The gender distribution was nearly balanced (51% male, 49% female), and the proportion of benign and malignant lesions was similar (49.2% vs. 50.8%), underscoring the heterogeneity of the included populations (Table 1). Considering the wide histopathological spectrum of soft tissue tumors, individual subtypes were not analyzed separately. The included studies differed in terms of histopathological composition, with variable distributions of benign lesions and sarcoma subtypes, which may have influenced reported elastographic values and contributed to inter-study heterogeneity.

### 3.2. Elastographic Findings

The selected studies employed different elastographic modalities, including strain elastography (SE), compression elastography (CE), and shear wave elastography (SWE). Three studies [13,16,21] used SE, reporting semiquantitative indices such as the strain ratio (SR) and elasticity score (ES). Seven studies [4,7,14,15,17,18,20] investigated SWE, presenting quantitative parameters such as shear wave velocity (SWV) or elasticity modulus values (kPa). The remaining studies applied qualitative elasticity scoring systems, such as the Tsukuba Elasticity Score (TES) or Park grading scale [5,16,19].

Across studies, elastography alone showed variable diagnostic accuracy, mainly due to methodological heterogeneity and operator dependence. Only five of the twelve studies demonstrated statistically significant differences in elastographic parameters between benign and malignant lesions [5,7,16,20,21].

As summarized in Table 2, malignant lesions generally exhibited higher stiffness values than benign ones. SWE-based parameters often yielded higher sensitivity, whereas SE-derived scores showed greater specificity when integrated with conventional ultrasound findings.

### 3.3. Ultrasound Systems and Study Conclusions

The technical characteristics of the ultrasound systems used, probe frequencies, and main conclusions of the included studies are presented in Table 3. The most frequently employed devices were the LOGIQ E9 (GE Healthcare) and Acuson S2000 (Siemens Medical Solutions). Probe frequencies ranged from 4 MHz to 18 MHz.

Despite heterogeneity in equipment and elastographic techniques, several consistent findings emerged:

Malignant tumors tended to exhibit higher stiffness and lower strain ratios than benign lesions [7,13,16].

SWE showed superior reproducibility compared with SE, owing to reduced operator dependency [17,20].

Multiparametric models combining elastographic indices with grayscale and Doppler ultrasound achieved the best diagnostic performance, with reported sensitivities and specificities exceeding 85% in some studies [5,21].

Clinical implication: Elastography was most effective when used as an adjunct to conventional imaging rather than as a stand-alone diagnostic tool, and may aid biopsy guidance by identifying the stiffest tumor regions [22].

## 4. Discussion

### 4.1. Diagnostic Performance of Elastosonography

Accurate imaging-based differentiation between benign and malignant soft tissue tumors (STTs) remains a complex diagnostic challenge. Although sonoelastography has been widely validated in hepatic fibrosis, breast, thyroid, and prostate lesions, its application in musculoskeletal oncology is still evolving [23,24,25,26,27]. The results of this systematic review confirm that ultrasound (US) elastography may provide additional diagnostic information beyond conventional imaging. Among the included studies, several authors reported significantly higher stiffness or strain ratios in malignant compared to benign lesions [7,13,16,20,21].

Riishede et al. [13] and Hahn et al. [16] demonstrated that the mean strain ratio was significantly higher in malignant tumors, confirming its potential role in predicting malignancy. Similarly, Li et al. [20] observed elevated Esd values and identified real-time shear wave elastography (rtSWE) patterns III–IV predominantly in malignant lesions. These findings support the hypothesis that increased tissue stiffness can serve as a surrogate indicator of malignancy.

However, other studies such as those by Bodard et al. [7] and Ozturk et al. [4] reported inconsistent results or limited statistical significance, particularly when elastography was used in isolation. This variability suggests that, while promising, elastography alone lacks sufficient accuracy to be a stand-alone diagnostic tool. The diagnostic performance of the technique appears to improve substantially when combined with grayscale and Doppler US findings. Composite diagnostic scores that integrate elastographic parameters have demonstrated the highest predictive values, with sensitivities and specificities exceeding 85% in some studies [5,21,28].

### 4.2. Technical Aspects and Methodological Variability

One of the most critical factors affecting diagnostic consistency across studies is the technical heterogeneity of elastosonographic methods. A comparative overview of the different elastographic technologies (strain/compression elastography vs. shear wave elastography), including qualitative and quantitative parameters and their diagnostic performance, is summarized in Table 2. Strain elastography (SE), compression elastography (CE), and shear wave elastography (SWE) are based on different physical principles, each with distinct advantages and limitations [4,7,10]. SE and CE are semi-quantitative, operator-dependent techniques relying on manual compression, whereas SWE provides a more objective and reproducible measurement of tissue stiffness by calculating the shear wave propagation velocity or elastic modulus.

Despite the theoretical superiority of SWE, variations in acquisition parameters, probe orientation, and measurement depth still introduce significant variability [17,18]. These technical inconsistencies and physical limitations have been comprehensively discussed in a recent review by Oglat et al. who emphasized the importance of standardizing acquisition settings and reporting criteria to improve reproducibility in clinical elastography [29]. Furthermore, the absence of standardized cutoff values for malignancy limits cross-study comparability. The elastic properties of tissues also depend on tumor composition—fibrosis, myxoid degeneration, necrosis, calcification, and cystic changes can all influence stiffness readings [7,18,30]. Consequently, high stiffness does not invariably indicate malignancy, nor does softness confirm benignity. In terms of operator repeatability, strain- and compression-based elastography are more susceptible to inter- and intra-operator variability, as measurements may be influenced by the amount and direction of applied compression, probe stability, and region-of-interest placement. In contrast, SWE is generally less operator-dependent and tends to show better repeatability; however, its measurements may still vary across ultrasound systems, acquisition protocols, and lesion depth. Importantly, only a limited number of the included studies reported formal repeatability metrics, such as intra- or inter-operator agreement, which currently limits a robust comparison of operator repeatability among different elastographic techniques.

Another challenge lies in the location of the lesion. Superficial STTs tend to yield more reliable elastographic data due to easier probe contact and reduced signal attenuation, whereas deep-seated or fat-containing lesions exhibit greater measurement variability [31]. Well-differentiated liposarcomas, for instance, may show low stiffness values similar to benign lipomas, complicating diagnosis [13,19]. Conversely, benign fibrotic or neural lesions, such as desmoid-type fibromatoses or schwannomas, can present unusually high stiffness, leading to potential false positives [32].

For these reasons, elastography should be interpreted as part of a multimodal approach, considering conventional ultrasound features, lesion morphology, and vascularity.

### 4.3. Clinical Integration and Implications

Although elastography cannot yet replace biopsy, its integration into clinical practice offers several potential advantages. As demonstrated by Bradley et al. [22], targeting the stiffest areas of a lesion during US-guided biopsy improved diagnostic yield by approximately 10%. This approach enhances histologic representativeness and reduces the number of inconclusive samples. In addition, elastography can support preoperative planning by delineating the most suspicious regions within a heterogeneous tumor.

Dou et al. [21] highlighted that combining elastography with patient age and grayscale ultrasound features yielded superior diagnostic performance (sensitivity 86%, specificity 91%) compared with any single parameter. Ohshika et al. [28] further confirmed the clinical potential of multiparametric ultrasound by integrating elasticity, vascularity, and tumor size into a three-item scoring system that achieved a sensitivity of 93.6% and specificity of 79.2%. Likewise, Yu Hu et al. [5] developed a clinic-ultrasonomics nomogram combining grayscale, color Doppler, and SE data, achieving an area under the curve (AUC) of 0.908–0.922 in predicting malignancy.

These findings collectively suggest that elastography functions best as a quantitative imaging biomarker when incorporated into a multiparametric diagnostic model. Beyond diagnostic differentiation, its ability to provide real-time mechanical characterization of tissue can support surgical planning and potentially influence the choice of treatment strategy in musculoskeletal and bone-adjacent lesions.

### 4.4. Limitations of Current Evidence

Despite encouraging data, several limitations restrict the generalizability of current findings. The main limitation is the small sample size and heterogeneity among the included studies. Most were single-center and observational, often with limited histologic diversity and unequal benign-to-malignant ratios. In addition, the histopathological composition varied substantially across studies, with different proportions of benign lesions and sarcoma subtypes. This heterogeneity did not allow a reliable comparison of elastographic performance according to specific tumor histotypes and may have contributed to inter-study variability. Methodological differences, including variable ultrasound systems, probe frequencies, and elastographic algorithms, also hinder direct comparison.

Operator dependency remains another critical issue, especially in strain-based techniques, where applied pressure and probe stability significantly affect measurements [16,17]. Additionally, the lack of uniform reporting standards—such as differences in ROI selection, measurement units (kPa vs. m/s), and threshold values—prevents the establishment of universally applicable diagnostic criteria.

Another source of bias stems from the overlap between soft tissue and bone tumors. Periosteal or bone-adjacent lesions may exhibit distinct mechanical properties that influence stiffness measurements [33]. In such cases, elastography should be carefully interpreted in combination with other imaging modalities such as MRI or CT. Finally, publication bias toward positive results may have led to overestimation of the technique’s performance in some studies.

### 4.5. Future Perspectives and Research Directions

Further research should focus on standardization and validation of elastosonographic techniques across centers. In this regard, Chowdhary et al. recently reviewed the expanding role of shear wave elastography in musculoskeletal imaging, highlighting its potential for longitudinal monitoring and integration with AI-based diagnostic tools [34]. Establishing reference cutoff values for stiffness and strain ratios specific to musculoskeletal tissues would enhance reproducibility. Integrating elastography with advanced imaging biomarkers—such as contrast-enhanced ultrasound (CEUS), diffusion-weighted MRI, or radiomics—could provide a more comprehensive understanding of tumor biology [35,36,37,38].

Building on this concept, Termite et al. recently proposed a standardized multiparametric ultrasound approach that combines morphological, vascular, and biomechanical parameters to optimize musculoskeletal tumor evaluation [8].

Moreover, the development of artificial intelligence (AI) and machine learning (ML) tools may soon enable automated lesion classification based on multiparametric ultrasound datasets. Preliminary studies suggest that combining elastographic features with AI-assisted pattern recognition could dramatically improve diagnostic accuracy and reduce operator dependency [5,28].

Ultimately, elastography should be viewed as an adjunctive technique within a broader imaging strategy that includes morphological, vascular, and biomechanical parameters. Its potential to refine preoperative risk stratification, reduce unnecessary biopsies, and improve diagnostic confidence aligns perfectly with the goals of personalized and precision oncology. In musculoskeletal and bone tumor management, elastosonography represents a promising quantitative imaging biomarker whose clinical utility is likely to expand as technology and analytical tools continue to evolve.

## 5. Conclusions

To the best of our knowledge, this is the first systematic review exploring the current state of elastosonographic applications in the characterization of soft tissue tumors (STTs). Although sonoelastography appears promising when integrated into a multiparametric diagnostic workflow, its standalone utility remains limited by technical variability and potential overlaps between benign and malignant soft tissue conditions.

The evidence reviewed clearly demonstrates that elastographic parameters, when interpreted in conjunction with grayscale and Doppler ultrasound findings, can enhance diagnostic confidence and accuracy. In particular, the inclusion of elastography within composite scoring systems has been shown to substantially improve both sensitivity and specificity in distinguishing benign from malignant lesions.

To date, no published studies have comprehensively evaluated the diagnostic potential of a fully multiparametric ultrasound approach combining B-mode, Doppler, elastography, and contrast-enhanced ultrasound (CEUS). Such an integrated model may represent the most effective strategy for improving the non-invasive differential diagnosis of soft tissue and bone-adjacent tumors in future clinical research.

Another important application of elastography is its proven ability to guide needle biopsy toward the most representative and biologically active tumor regions, thereby improving sampling accuracy and reducing nondiagnostic results.

While current evidence does not support replacing biopsy as the diagnostic gold standard, future studies are expected to refine the clinical value of elastosonography, leading to faster and more accurate diagnoses, fewer unnecessary invasive procedures, and improved management of patients with musculoskeletal and bone-related soft tissue tumors.

## Figures and Tables

**Table 1:** Demographic and clinical characteristics of included studies. Data are presented as mean (range) where available.

Year	Study (Author, Ref.)	Study Type	Patients (n)	Mean Age (Years)	Gender (M:F)	Tumor Behavior (Benign:Malignant)
2022	Bodard et al. [7]	Prospective	136	49.3 (16–86)	69:67	54:82
2015	Riishede et al. [13]	Prospective	60 (61 tumors)	–	27:33	42:19
2022	Yu Hu et al. [5]	Mixed (prospective + retrospective)	270	–	108:140	169:89
2016	Pass et al. [14]	Prospective	105	52.3 (20–88)	57:48	66:39
2016	Pass et al. [15]	Prospective	50	56 (18–85)	33:17	35:15
2016	Hahn et al. [16]	Retrospective	73	50 (5–79)	36:37	40:33
2019	Tavare et al. [17]	Prospective	206	57.7 (18–91)	89:117	127:69
2020	Winn et al. [18]	Prospective	148	54.5 (18–91)	76:72	87:61
2022	Cohen et al. [19]	Retrospective	137	–	80:57	81:56
2020	Li et al. [20]	Prospective	73 (81 tumors)	43.9 (11–84)	30:43	60:21
2020	Ozturk et al. [4]	Prospective	109	43.3 (0–85)	64:45	72:37
2021	Dou et al. [21]	Prospective	83	49.8 (18–85)	43:40	47:36

**Table 2:** Summary of elastosonographic findings across included studies.

Study (Author, Ref.)	Elastographic Modality/Parameter	Mean Value (Benign)	Mean Value (Malignant)	*p*-Value	AUC	Sensitivity (%)	Specificity (%)	PPV (%)	NPV (%)	Main Findings
Bodard et al. [7]	SWE (kPa)	32.1	51.2	0.004 *	–	50 (cut-off 40.8 kPa)	79 (cut-off 40.8 kPa)	–	–	Malignant lesions show higher stiffness values.
Bodard et al. [7]	SWE (T/F ratio)	2.34	4.10	0.047	–	46 (cut-off 3.5)	84 (cut-off 3.5)	–	–	Tissue-to-fat ratio increases in malignancy.
Riishede et al. [13]	Strain Ratio (SR)	1.35	1.94	0.043	–	–	–	–	–	SR significantly higher in malignant tumors.
Yu Hu et al. [5]	Tsukuba Scoring System (1–5)	18/34/24/19/1	1/6/21/17/7	<0.001 *	–	–	–	–	–	Higher elasticity scores correlated with malignancy.
Pass et al. [14]	SWE (SWV)	–	–	NS	–	–	–	–	–	No significant difference in SWV.
Pass et al. [15]	SWE (Longitudinal SWV, m/s)	2.20	1.59	0.095	–	–	–	–	–	Trend toward lower SWV in malignant lesions.
Hahn et al. [16]	Elasticity Score (Itoh)	3.08 ± 1.44	3.76 ± 0.97	0.048	0.623	–	–	–	–	Malignant lesions show higher ES.
Hahn et al. [16]	Strain Ratio (SR)	1.03 ± 0.93	0.49 ± 0.45	0.003 *	0.700	–	–	–	–	SR useful to predict malignancy.
Tavare et al. [17]	SWE (SWV)	–	–	0.87	0.50	93	72	31	99	SWE improves diagnostic classification vs. US alone.
Winn et al. [18]	SWE (SWV, m/s)	4.36	3.73	0.06	–	–	–	–	–	Trend toward slower SWV in malignant lesions.
Cohen et al. [19]	Strain Ratio (SR)	2.30	2.66	0.30	–	–	–	–	–	No significant difference in SR.
Cohen et al. [19]	Tsukuba Elasticity Score (TES)	3.16	3.49	0.043	–	84 (TES 3)	24 (TES 3)	56 (TES 4)	57 (TES 4)	TES ≥ 3 associated with higher malignancy rate.
Li et al. [20]	rtSWE (Esd, kPa)	0.42	0.88	<0.001 *	–	71.4	66.7	85	60.9	Higher Esd values in malignant tumors.
Li et al. [20]	rtSWE (Pattern III–IV)	1/5	2/11	<0.001 *	–	69.2	61.9	90	68.4	Patterns III–IV predictive of malignancy.
Ozturk et al. [4]	SWE (SWV mean, m/s)	2.94 ± 1.5	3.57 ± 2.25	0.271	–	–	–	–	–	No significant difference in SWV.
Dou et al. [21]	Elasticity Score (ES < 2.5)	2.36 ± 0.82	2.81 ± 0.71	0.011	0.662	69.4	63.8	–	–	ES significantly higher in malignant lesions.
Dou et al. [21]	EI/B ratio (>1)	31	14	<0.001 *	0.782	–	–	–	–	Combined SE and US improved diagnostic accuracy.

Abbreviations: SWE, shear wave elastography; SE, strain elastography; SR, strain ratio; SWV, shear wave velocity; TES, Tsukuba Elasticity Score; rtSWE, real-time shear wave elastography; Esd, elasticity standard deviation; EI/B, elastography-to-B-mode lesion size ratio; T/F, tissue-to-fat ratio; PPV, positive predictive value; NPV, negative predictive value; AUC, area under the curve; Asterisks (*) indicate statistical significance (*p* < 0.05). “NS”: not statistically significant.

**Table 3:** Ultrasound systems, probe characteristics, and main conclusions from included studies.

Study (Author, Ref.)	Ultrasound System	Probe Frequency (MHz)	Study Duration	Main Conclusions
Bodard et al. [7]	APLIO 500 (Canon Medical Systems, Otawara, Japan)	14	May 2015–Oct 2016	SE highlighted higher elasticity and T/F values in malignant STTs compared with benign ones.
Riishede et al. [13]	LOGIQ E9 (GE Healthcare, Chalfont St Giles, UK)	9L, ML6–15, C1–5	3 months (not specified)	SR significantly higher in malignant tumors; potential adjunct in STT diagnosis.
Yu Hu et al. [5]	LOGIQ E9 (GE Medical Systems, Wauwatosa, WI, USA)	L6–15, C1–6	Apr 2018–Oct 2020	SE combined with grayscale US, CDFI, and clinical data in a multiparametric nomogram predicted malignancy with high accuracy.
Pass et al. [14]	Acuson S3000 (Siemens, Erlangen, Germany) with ARFI (Virtual Touch Quantification)	9–4	Not specified	SWE measurements did not improve malignancy detection.
Pass et al. [15]	Acuson S2000 (Siemens AG, Erlangen, Germany) with ARFI	9–4	Not specified	SWE showed trends but limited evidence for malignancy correlation.
Hahn et al. [16]	Acuson S2000 (Siemens Medical Solutions, Mountain View, CA, USA)	18L6HD	Apr 2012–Oct 2014	SR useful diagnostic indicator for predicting malignancy in STTs.
Tavare et al. [17]	LOGIQ E9 (GE Healthcare)	9–4	Dec 2015–Mar 2017	SWE may supplement conventional US for improved classification of STTs.
Winn et al. [18]	Acuson S2000 (Siemens)	9–4	May 2016–Nov 2017	SWE showed slower velocities in malignant lesions; histology remains gold standard.
Cohen et al. [19]	LOGIQ E9 (GE Healthcare)	9L, ML6–15, C1–5	May 2014–May 2016	SE may be a valuable adjunct to B-mode US for primary diagnostic assessment.
Li et al. [20]	Aixplorer (Supersonic Imagine, Aix-en-Provence, France)	L4–15, C1–6	Sep 2016–Jan 2020	SWE provides a useful non-invasive method for predicting malignancy in STTs.
Ozturk et al. [4]	Acuson S2000 (Siemens Medical Solutions, Mountain View, CA, USA)	L9	Aug 2016–Jan 2020	SWE not discriminatory for malignancy prediction in STTs.
Dou et al. [21]	LOGIQ E9 (GE Healthcare, Wauwatosa, WI, USA)	9L, ML6–15, C1–5	May 2019–Oct 2020	Combining SE with conventional US improved diagnostic performance for malignant STTs.

Abbreviations: US, ultrasound; CDFI, color Doppler flow imaging; SE, strain elastography; SWE, shear wave elastography; SR, strain ratio; ARFI, acoustic radiation force impulse; STT, soft tissue tumor; MHz, megahertz.

## Data Availability

No new data were generated or analyzed in this study.

## Supplementary Materials

The following supporting information can be downloaded at: https://www.mdpi.com/article/10.3390/jcm15020498/s1, Table S1: PRISMA Checklist. Reference [39] is cited in the Supplementary Materials.
